# Unusual Vasospastic Angina: A Documented Asymptomatic Spasm with Normal ECG—A Case Report and a Review of the Literature

**DOI:** 10.1155/2013/407242

**Published:** 2013-04-22

**Authors:** Ghassan Nakad, Hamid Bayeh

**Affiliations:** ^1^Lebanese University, Beirut 6573, Lebanon; ^2^Notre Dame des Secours University Hospital, Holy Spirit University, Joubeil 1401, Lebanon

## Abstract

We report the case of a 53-years-old patient, known to have coronary artery disease, presenting with typical angina at rest with normal ECG and laboratory findings. His angina is relieved by sublingual nitroglycerin. He had undergone a cardiac catheterisation two weeks prior to his presentation for the same complaints. It showed nonsignificant coronary lesions. Another catheterisation was performed during his current admission. He developed coronary spasm during the procedure, still with no ECG changes. The spasm was reversed by administration of 2 mg of intracoronary isosorbide dinitrate. Variant (Prinzmetal's) angina was diagnosed in the absence of electrical ECG changes during pain episodes.

## 1. Background

Coronary spasm is defined as a condition in which a relatively large coronary artery exhibits abnormal contraction (spasm). If the spasm induces a complete or nearly complete occlusion, transmural ischemia occurs in the region perfused by the artery, which in turn causes angina attacks with ST elevation on the ECG. If a partial occlusion occurs, or a sufficient collateral flow has developed distally, nontransmural ischemia occurs, causing anginal attacks with ST depression on the ECG. 

These pathological conditions are collectively termed vasospastic angina (also termed coronary spastic angina), as a type of angina pectoris caused by coronary spasm [1].

Variant angina pectoris, characterized by ST elevation during anginal attacks, is a type of vasospastic angina. Variant angina (Prinzmetal's angina or periodic angina) is a form of unstable angina that usually occurs spontaneously and is characterized by transient ST-segment elevation that spontaneously resolves or resolves with nitroglycerin (NTG) use without progression to myocardial infarction, usually in the presence of coronary artery disease. Clinically, the patient presents with chest discomfort, mostly at rest without any preceding increase in myocardial oxygen demand [2].

The pathogenesis relies upon focal coronary artery spasm, in a single or multiple vessels, leading to transient severe transmural myocardial ischemia.

In the present guidelines, the diagnostic criteria for vasospastic angina are established for three grades: “definite,” “suspected,” or “unlikely” [1]. Those criteria are provided in the discussion section. Typically, NTG is exquisitely effective in relieving the spasm. Coronary angiography is usually part of the workup of these patients and can help orient treatment [2].

## 2. Case Presentation

**Figure 1:**
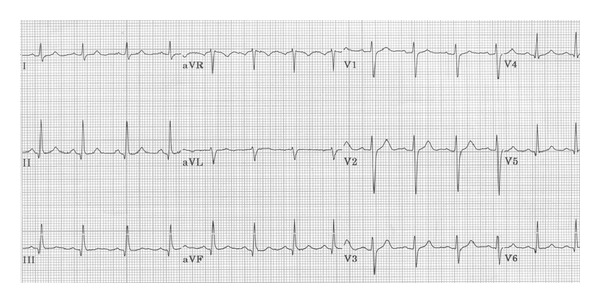
ECG during pain crisis.

A 53-year-old male is admitted late morning for severe chest pain, with interscapular irradiation, associated with nausea and diaphoresis. He had been complaining of the same pain for a month and a half prior to presentation. Symptoms occurred sporadically during the day, lasting from several minutes to sometimes a bit less than an hour, at rest with no association with effort. He is known to have coronary artery disease (CAD), for which he underwent percutaneous angioplasty (PTCA) 1.5 years earlier on a tight lesion of the proximal LAD. A cardiac catheterization was performed 2 weeks prior to admission for the same complaints and revealed nonsevere coronaries lesions. We note that the patient reported the same quality of pain prior to his PTCA with relief of symptoms after revascularization at that time. An electrocardiogram (ECG) was performed during pain episode and was normal with no ST-segment changes (Figure 1). Due to the high clinical suspicion of an angina and the low clinical probability of alternative diagnosis, the patient was given 5 mg of sublingual NTG, resulting in complete relief of symptoms after about 5 minutes. Cardiac enzymes were drawn and reported as normal, with no other significant blood analysis findings. The patient was admitted to the hospital for investigations.

Several hours after admission, the patient suffered from another identical episode of chest pain, with normal ECG during the pain crisis, and again pain was relieved after administration of 5 mg of sublingual nitroglycerin. Cardiac enzymes were also reported as negative 8 hours after admission.

Subsequently, a cardiac catheterization was opted for, due to high suspicion of an acute coronary syndrome (ACS), keeping in mind that our patient had already undergone a catheterization 2 weeks earlier.

**Figure 2:**
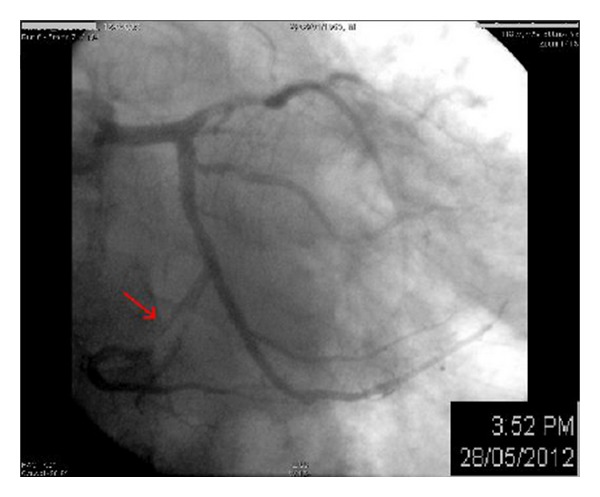
Coronarography showing a near total occlusion of the circumflex artery (red arrow).

**Figure 3:**
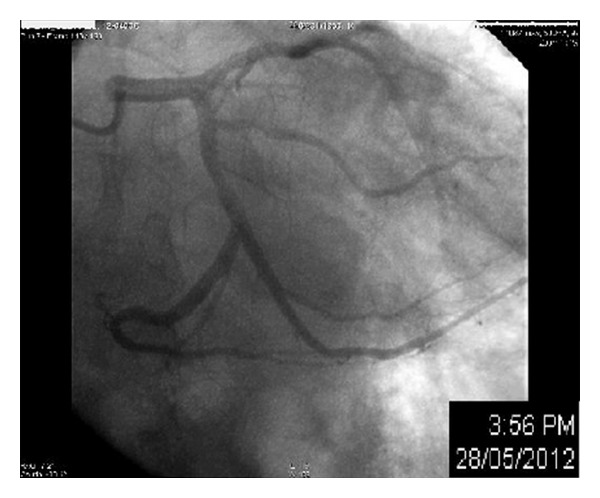
Relief of the stenosis after intracoronary NTG.

Coronarography was performed, showing nonstenotic coronary lesions at first, but after several contrast dye injections, the atrioventricular (A-V) groove branch of the circumflex artery showed a near total occlusion. The patient was asymptomatic at that time, and no ST-segment changes were noted the continuous ECG monitor in the catheterization lab. An angioplasty was elected for to revascularize the critical lesion. A guiding wire was inserted into the left coronary, and 2 mg of intracoronary NTG were given before further advancing the wire. This led to a complete relief of the previously detected lesion; so, the procedure was halted, and the diagnosis of vasospastic angina (VSA) was retained (Figures 2 and 3).

The patient was discharged on maximal dose of calcium channel blockers (CCBs) and sublingual NTG as needed. He was followed up at 2 weeks and then after a month with total resolution of symptoms.

## 3. Case Discussion

The controversies in this case rely on two facts: first and most important, the presence of a documented coronary spasm, with neither concomitant symptoms nor ECG changes on the catheterization lab monitor, and second, the repeatedly normal ECGs during pain episodes.

The diagnosis of VSA is categorized into “definite, suspected, and unlikely.”

A definite diagnosis is made in the presence of anginal symptoms and ischemic ECG changes (transient ST elevation of 0.1 mV or more, ST depression of 0.1 mV or more, or new emergence of negative U waves, recorded in at least two contiguous leads on the 12-lead ECG) or a clear finding of myocardial ischemia or coronary spasm obtained on examinations (drug-induced coronary spasm provocation test during cardiac catheterization and hyperventilation test) in relation to the symptoms in the setting of a borderline ECG [1].

A suspected VSA is counted for in the presence of symptoms associated with a borderline ECG but no clear finding of myocardial ischemia or coronary spasm in any examination or a negative ECG associated with the following features: an attack that resolves quickly on administration of NTG and either appears at rest particularly between night and early morning or has a marked diurnal variation in exercise tolerance (in particular, reduction of exercise capacity in the early morning) or is induced by hyperventilation (hyperpnea) or the fact that those attacks are suppressed by CCBs but not by *β*-blockers [1].

VSA is unlikely in the setting of a normal ECG and if the previous features are not present [1].

Asymptomatic angina has been described. It is defined as typical ECG changes recorded on an ambulatory ECG monitoring, depicting underlying ischemia, with absence of angina symptoms [3].

The dilemma resides in the fact that our patient had a documented vasospasm with neither symptoms nor ECG changes and several normal ECGs at time of pain attacks during his hospitalization. Those attacks were relieved by NTG and in retrospect by CCBs after discharge. If no spasm was detected, our patient would have been classified as unlikely, or at best if an arbitrary trial of CCBs was to be started, he would have been classified as suspicious of VSA.

Moreover, the current published guidelines do not allow us to reach a definite diagnosis as our patient had neither positive ischemic ECG signs, nor symptoms at the time of the documented spasm or at the time of pain. 

The danger resides in the eventuality of wrongfully stenting the spasm (if no NTG was to be given), assuming that it is a lesion with adequate collateral flow explaining the absence of ischemic symptoms, or at best if the spasm was not detected, to fail to diagnose and treat a VSA.

Prolonged vasospasm might lead to complications such as myocardial infarction (MI), high degree of A-V block, ventricular tachycardia, and sudden death [4–7].

We reviewed the literature in order to understand the atypical ECG presentation. Several cases were reported depicting alternative ECG changes representative of ischemia in VSA. ST segment depression was reported in Prinzmetal's angina [8], which can represent a subendocardial ischemia in contrast to the classical transmural ischemia. That might be partly due to the presence of collateral circulation through preexisting vessels to the ischemic region caused by the vasospasm [9]. The documentation of such collaterals remains a challenge because of the technical difficulties in the simultaneous visualization of the spastic and nonspastic arteries responsible for the collateral flow [10].

In our case, no ECG changes were detected in spite of a typical clinical presentation. Two suggestions arise. The first is that the electrical changes of the anatomical territory supplied by the A-V groove branch (posterior wall) might not be readily identified on the 12-lead ECG [11]. The second explanation relies on the fact that a TIMI flow of 3 was detected when the vasospasm was underway. This might be due to an incomplete spasm and to a collateral circulation that probably resulted in modest myocardial ischemia.

 In fact, Tasaki et al. reported a case of variant angina with no ST segment modifications [12]. In that case, a recruitable backflow was detected both in systole and diastole, using a Doppler guide wire place in the right coronary artery after inducing a spasm by injection of Ergonovine Maleate [10].

## 4. Implications for Treatment

This case shows an unusual presentation of vasospastic angina. The diagnostic challenge is posed by the incidentally documented spasm that failed to produce any symptoms or ECG changes at time of catheterization and the negative ECG findings during the acute episodes. 

Coronary provocative tests are controversial if no ECG is available during the episode, and thus no ECG documentation is present in the setting of a high clinical suspicion (level of evidence IIb,C) and is contraindicated if Prinzmetal's angina is diagnosed by ECG (level of evidence III,B) [1].

No guidelines are available regarding the presence of a negative ECG in the setting of a high clinical suspicion (but not meeting the afore mentioned criteria in “suspected VSA”). Furthermore, Prinzmetal's angina occurs mostly on apparently healthy coronary segments of people with preexisting CAD, also posing a contraindication for provocative testing if high degree coronary stenosis is present (level of evidence III,B) [1].

None of the available guidelines discusses the management of an asymptomatic, negative ECG spasm. Due to our high clinical suspicion in spite of a normal ECG and to the occurrence of the spasm even if asymptomatic, we opted for a conventional therapy as for VSA.

Our approach was to administer a treatment consisting of a standing dose of CCB and NTG as needed. Judgment was made according to patient's response.

The cornerstone treatment of coronary spasm is NTG. Long acting nitrates and CCBs are considered first-line therapies. Beta-blockers have theoretical adverse potential, and their clinical effect is controversial. Smoking should be discontinued. Patients with very active disease can require a combination of nitrates and CCBs. Alpha-receptor blockers have been reported to be of benefit [1].

Some patients may require a pacemaker or a defibrillator to prevent transient AV block or to prevent sudden death [1, 4, 5]. In patients refractory to standard medication, cardiac denervation might be attempted with marginal benefit [1].

The prognosis of variant angina is usually excellent in patients who receive medical therapy, especially in patients with normal or near-normal coronary arteries [1].
